# Challenges in the Early Diagnosis, Screening and Management of Heart Failure in Patients with Chronic Obstructive Pulmonary Disease

**DOI:** 10.3390/jcm15082978

**Published:** 2026-04-14

**Authors:** Roop Kaw, Aniruddh S. Shah, Shashank Shekhar, Michael Faulx, Loutfi S. Aboussouan

**Affiliations:** 1 Departments of Hospital Medicine, Outcomes Research, Anesthesiology, Integrated Hospital Care Institute, Cleveland Clinic, Cleveland, OH 44195, USA; 2 Department of Pulmonary and Critical Care Medicine, Respiratory Institute, Cleveland Clinic, Cleveland, OH 44195, USA; shaha13@ccf.org; 3 Harrington Heart and Vascular Institute, University Hospitals Cleveland Medical Center, Cleveland, OH 44195, USA; shashank.shekhar@uhhospitals.org; 4 Section of Clinical Cardiology, Heart, Vascular and Thoracic Institute, Cleveland Clinic, Cleveland, OH 44195, USA; faulxm@ccf.org; 5 Department of Pulmonary and Critical Care Medicine, Respiratory Institute, Sleep Disorders Center, Neurologic Institute, Cleveland Clinic, Cleveland, OH 44195, USA; aboussl@ccf.org

**Keywords:** chronic obstructive pulmonary disease, congestive heart failure

## Abstract

In patients with co-morbid CHF and COPD, the diagnosis of CHF can be delayed. It is also well known that left ventricular dysfunction can arise from progressive disease-related hyperinflation. Apart from the longitudinal risk of developing CHF in some patients, a short-term or subclinical risk of cardiac events has been reported after hospitalization for COPD exacerbation. Currently there are no data or strategies to support screening for the early diagnosis of CHF in patients with COPD. Similarly, pulmonary function testing results can also be confounding and inaccurate in establishing the severity of COPD during an active exacerbation of CHF. The hyperinflation of the lungs, which can alter LV geometry and mechanics, is at the root of many of the causes of LV underfilling, which eventually contributes to CHF. Conventional echocardiography can often miss subclinical myocardial dysfunction and hence make early diagnosis even more challenging. Advanced cardiac imaging modalities and revised echocardiographic parameters can help detect subclinical LV dysfunction and PH earlier, but there are no clinical outcome data to validate their routine use in day-to-day clinical practice. Beta-blockers are generally regarded as safe to be used for appropriate cardiovascular indications in patients with COPD, and recent trials have also established the safety of long-acting beta agonists for treating COPD in patients with elevated cardiac risk.

## 1. Introduction

Congestive heart failure (CHF) frequently goes undetected in patients admitted for acute exacerbations of chronic obstructive pulmonary disease (COPD) [1]. Although CHF and COPD may have common etiologic risk factors and may be co-morbid, evidence from a Mendelian randomization study and a prospective population-based study suggest that COPD may contribute to the development of CHF and that this relationship is not bidirectional [2,3]. While these studies do not suggest causality, they emphasize the potential importance of COPD identification and management in the prevention of heart failure. Besides common etiologic risk factors, complex cardiopulmonary interactions arising from the age-related decline in lung function and type of co-morbid lung disease can result in heart failure in many such patients. Many similarities in the clinical presentation can hinder early diagnosis and often result in diagnostic errors when such patients require hospitalizations.

## 2. Burden of Co-Morbid CHF in COPD

### 2.1. Scope of Problem

The prevalence of systolic or diastolic heart failure among patients with COPD has been reported to range from 20 to 70% with an annual incidence of 3–4% [4]. In a large sample of the collaborative general practices of 370,000 registered patients, a 3-fold increase in heart failure and 1.7-fold increase in ischemic heart disease was noted in patients with COPD [5]. Left ventricular dysfunction has been identified in 40% of patients treated with mechanical ventilation for hypercapnia failure resulting from COPD [6].

### 2.2. Outcomes of COPD Patients with Co-Morbid CHF

Population studies of patients with COPD and no history of cardiovascular disease have reported a 25% increase in the adjusted risk of major adverse cardiac events, which include acute myocardial infarction, stroke and cardiovascular death [7]. To provide perspective, this compares to an 18% risk-adjusted cardiovascular mortality from diabetes and 31% risk-adjusted cardiovascular mortality from CKD, but despite this, COPD is not included in traditional cardiovascular risk prediction models. Cardiovascular deaths are more commonly reported than respiratory deaths among patients with mild or moderate COPD classified per a GOLD report [8]. In addition, co-morbid CHF leads to higher all-cause and HF-specific hospitalizations, including first heart failure hospitalization, also reported among patients hospitalized with COPD exacerbations. From a phenotype standpoint, although both HFpEF and HFrEF can lead to HF-specific hospitalizations in patients with COPD, this risk was noted to be higher among those with co-morbid HFrEF [9]. Several important studies reporting these outcomes are outlined in Table 1.

## 3. Pathophysiology

### Cardiopulmonary Interactions in Patients with COPD

Cardiopulmonary interactions are complex. One of the ways to understand the heart–lung interaction is through static (loss of elastic recoil due to hyperinflation) and dynamic lung hyperinflation (prolonged expiration with incomplete emptying from bronchial obstruction), which is a core functional abnormality in COPD. In established COPD, the extent of airflow obstruction by spirometry and emphysema by CT scan have been shown to be linearly related to impaired LV filling and decreased cardiac output without a change in ejection fraction [19]. Patients with emphysema suffer a relatively larger loss of alveolar surface and therefore hyperventilate both at rest and with exercise, which also helps maintain their SaO_2_ and PaCO_2_ within normal ranges compared to those with predominant chronic bronchitis. The associated severity of pulmonary vascular loss also contributes to reduced pulmonary venous return and subnormal cardiac output, and therefore heart failure is seen more terminally in patients with emphysema compared to those with chronic bronchitis [20]. In chronic bronchitis, contrarily, relatively lower PaO_2_ levels provide the stimulus for hypoxic pulmonary vascular constriction, higher PVR and earlier evidence of heart failure [21]. Improvements in right and left ventricular stroke volumes have been reported with the concomitant use of the corticosteroids, beta-2 agonists and anticholinergics used to treat hyperinflation in patients with COPD [22]. The differential patterns of decline in FEV1/FVC (with and without preserved FEV1/FVC ratio) from peak lung function in otherwise healthy young adults to that in middle-aged adults have been shown to be associated with a hypertrophic high-output systolic failure phenotype or underfilled, small heart phenotypes, respectively, even before the onset of COPD or CHF [23]. More importantly, it is widely believed that subclinical LV dysfunction can be associated with the degree of severity of COPD even in the absence of major LV structural alteration, LV or RV dysfunction or PH.

## 4. Onset of CHF and Screening in Patients with COPD

### 4.1. Case for Early Screening of CHF

Numerous studies reveal that the risk of adverse cardiovascular events (arrhythmia, heart failure, acute coronary syndrome, stroke) peaks from 14 to 30 days after an acute exacerbation and, in many instances, lasts for 12–18 months compared to those without any exacerbation of COPD [24,25]. The risk of incident atrial fibrillation (AF) during COPD exacerbations has been reported to be 28% [26] and shown to correlate inversely with FEV1 [27]. These adverse cardiovascular events were reported regardless of whether these patients were at any risk for cardiac events [28,29], frequency of prior COPD exacerbations [30] or randomized treatment [29]. This establishes the basis for screening for undiagnosed CHF in patients with COPD [31], although it is unclear whether such screening can be cost-effective or even practical in patients with stable COPD or after an exacerbation [32].

### 4.2. Value of NT-ProBNP in Screening for HF in Patients with COPD

The levels of NT-proBNP have been reported to rise during acute exacerbations of COPD [33]. Therefore, its role in screening for HF in patients hospitalized with an acute exacerbation of COPD (AECOPD) is unclear. Using transthoracic echocardiography (TTE) data in an unselected cohort within 100 days of AECOPD in many cases (50%) with time-matched NT-proBNP (>400 pg/mL) drawn within 7 days of TTE, over 40% of new cases of HF were diagnosed [15]. The selected cut-off for NT-proBNP chosen to avoid confounding from AECOPD and acute illness yielded an NPV of 77.8% and PPV of 82.8%, suggesting the role of screening in patients admitted with AECOPD; however more data is awaited.

Elevated NT-proBNP levels have also been reported to be an early (30 days) and late predictor of mortality (5 years) among patients with COPD exacerbation [34] regardless of any known or pre-existing heart disease. Comparatively, troponin elevations were noted to be associated only with short-term mortality, suggesting that unless these elevations meet the bar of MI/ischemia, they do not help quantify any cardiac remodeling/long-term dysfunction that the elevations in NT-proBNP may help unravel. The elevation in NT-proBNP happens during the first few days of COPD exacerbation and falls after a few weeks after recovery and is also predictive of readmission related to cardiac dysfunction and not COPD exacerbation.

### 4.3. When and Why of Pulmonary Function Testing in Patients with Heart Failure

In a cluster-randomized trial of 386 frail patients, >65 years of age, that excluded patients with an established diagnosis of COPD+HF and only included those with either a diagnosis of COPD or HF or symptoms of dyspnea or reduced exercise tolerance, Bertens and colleagues reported that nearly 21.8% of screened patients received a new diagnosis of COPD, although this had little impact on the management of these patients [35]. This further illustrates the viability and efficacy of screening symptomatic patients for the presence of COPD. The European Society of Cardiology’s guidelines on HF recommend considering screening with pulmonary function testing using spirometry in suspected COPD patients [36]. Correctly interpreting the spirometry results in patients with CHF may be challenging, since the test cannot be performed in patients with rapidly increasing decompensation, thereby leading to an overdiagnosis of COPD [37]. Diffusing capacity combined with NT-proBNP can sometimes help in wet lung diagnosis as long as it is understood that some if not most patients with dry lung HF can have a reduced DLCo from damage to the alveolar–capillary membrane in the absence of vascular congestion or COPD [38].

## 5. Diagnostic Challenges in Patients with COPD and CHF

### 5.1. Limitations of Pulmonary Function Testing

In the context of acute decompensated heart failure (ADHF), pulmonary function testing has no specific diagnostic, prognostic or therapeutic application unless it is necessary for qualifying purposes such as the initiation of home non-invasive ventilation. However, abnormalities on testing are highly prevalent. In a 2021 study, Kawakami et al. evaluated a cohort of 1012 patients admitted with ADHF, of whom 657 patients underwent spirometry prior to discharge. Abnormal PFTs were noted in 63% of patients with restrictive patterns being the most common (36.7%), followed by mixed and obstructive patterns. Notably, only about 30% of patients with abnormal PFTs had any documented lung disease, suggesting the involvement of cardiac congestion in these ventilatory abnormalities rather than pre-existing pulmonary pathology [39].

The following patterns of abnormalities have been reported on PFTs among heart failure patients based on the degree and distribution of volume overload.

*Restrictive pattern mimicry:* Interstitial lung edema associated with chronic HF reduces pulmonary compliance, leading to a restrictive pattern in which PFT abnormalities primarily mirror the degree of fluid overload and related pulmonary vascular disease, rather than intrinsic lung parenchymal disease [40]. This combination leads to concurrent reductions in both the forced vital capacity (FVC) and FEV1. Pleural effusions can independently induce restrictive changes with a proportional reduction in lung volume parameters like TLC and FVC. Obesity, a frequent co-morbidity in HF, can further impose additional extrathoracic mechanical load on the diaphragm and chest wall, accentuating the restrictive physiology [41].

*Pseudo-obstructive physiology:* The accumulation of interstitial and peribronchial fluid from venous hypertension in heart failure can compress small airways, creating a transient airflow limitation [42]. In some cases, true bronchial flow limitation arises from mucosal edema due to bronchial congestion or extrinsic airway compression caused by reduced intrathoracic space secondary to cardiac enlargement and pulmonary edema [43,44]. This combination leads to concurrent reductions in both the forced vital capacity (FVC) and the forced expiratory volume in 1 s (FEV1), with a disproportionately greater reduction in FEV1, resulting in an obstructive ventilatory pattern [39]. Such transient obstruction can be misinterpreted, risking a false-positive diagnosis of COPD when FEV1/FVC appears reduced. For instance 19% of patients had airway obstruction on spirometry at discharge following hospitalization for systolic heart failure, but this obstructive impairment resolved in 47% at re-evaluation in the stable state 6 months later [45]. Hence, the spirometry performed during episodes of acute decompensated heart failure should not be used to diagnose COPD, and it is emphasized that PFTs should be performed after the stage of heart failure decompensation is over or treated.

*Diffusion impairment:* The extravasation of fluid into the lung interstitium and alveoli reduces gas diffusion efficiency by widening the barrier through which oxygen and carbon monoxide must traverse to reach capillary blood. This process compromises the overall diffusing capacity of the lungs. Minasian et al. demonstrated that 44–58% of outpatient heart failure patients exhibited a DLCO below the conventional lower limit of normal [38]. From a diagnostic standpoint, a reduction in the DLCO in the setting of decompensated heart failure typically reflects reversible pulmonary edema or congestion, rather than a primary, irreversible parenchymal abnormality such as true emphysema [43].

#### 5.1.1. “Wet” Versus “Dry” Lung States in Patients with Heart Failure

PFTs performed during the dry lung state offer a more accurate reflection of intrinsic, irreversible lung disease. While the total lung volumes, including FEV1 and FVC, may remain reduced (often by about 10% to 20% in HFrEF, compared to controls), in dry lung HF, these reductions are typically proportional, and FVC and the DLCO are normal [32]. However, in chronic heart failure, permanent damage to the alveolar–capillary membrane can result in a reduced DLCO even in the absence of volume overload or emphysema [38].

In wet lung heart failure, both FEV1 and FVC decrease, while FEV1/FVC may be proportionately normal (restrictive) or reduced (pseudo-obstructive), and the DLCO is reduced. These patients were also found to have 25% lower pulmonary artery compliance and 25–35% higher pulmonary vascular resistance and pressures when compared to “dry lung” patients [46]. Importantly, the pseudo-obstruction seen in the wet state may improve or even normalize after successful decongestion. When clinical stability is uncertain, body plethysmography-derived measurements, such as the ratio of residual volume to total lung capacity (RV/TLC), are preferred for the diagnosis of COPD, as they are considered reliable indicators of air trapping and are not influenced by pulmonary congestion [40]. While these functional abnormalities often improve with decongestion, prolonged pulmonary congestion with recurrent decompensation episodes in these patients may lead to structural airway and parenchymal remodeling. Over time, this can cause bronchial wall thickening and fibrotic alterations, eventually resulting in irreversible restriction with a reduced TLC and, to a lesser extent, FEV1, thus making it difficult to correctly classify the severity of COPD in the presence of co-morbid CHF [47].

The physiological consequence of these interactions is that PFT abnormalities in these patients often serve as markers of the severity of cardiac hemodynamics, correlating with parameters such as elevated pulmonary artery systolic pressure (PASP) and left ventricular mass (LVM), which may occur even before the clinical signs of cardiac decompensation manifest [48]. As a result, abnormal PFT results may reflect the degree of cardiac-induced congestion rather than intrinsic pulmonary pathology. This makes it difficult to use PFTs to confidently differentiate whether the patient’s impairment is predominantly cardiac-induced congestion or a pre-existing, non-HF-related lung disorder.

#### 5.1.2. Prognostic Value of PFTs in Patients with Heart Failure

Despite the interpretive challenges, PFTs’ still offer significant and independent prognostic information regarding disease progression and mortality in these patients. A lower FEV1 was associated with worsening HF (stages B through D), independent of confounders like COPD [49]. New-onset HFpEF patients in an outpatient study with airflow limitations were found to have a higher all-cause mortality compared to those with normal spirometry [50]. In stable chronic heart failure cohorts, impaired alveolar–capillary membrane conductance (<24.7 mL/min/mmHg) (DM), a component of DLCO, was also found to be an independent predictor of worse prognosis and cardiac death [51]. Similarly, a restrictive spirometric pattern in these stable HF patients is independently associated with increased mortality as well, with mixed obstructive–restrictive patterns suggesting a particularly poor prognosis. Importantly, these prognostic associations are applicable mainly to heart failure patients with ventilatory defects in the absence of concomitant pulmonary hypertension [52]. In advanced HFpEF patients complicated by pulmonary hypertension and high pulmonary pressures, a significantly low DLCO was noted to be associated with increased mortality [53]. The alveolar volume measured during DLCO tests has also been shown to predict mortality in patients with HFrEF [54].

### 5.2. Limitations of Echocardiography in COPD and COPD-HF Overlap

Despite being first-line and easily available for screening, transthoracic echocardiography has reduced feasibility and accuracy in COPD and can miss early disease. In these patients, hyperinflation-related acoustic dropout and respiratory pressure swings foreshorten apical views, distort Doppler signals, and confound conventional metrics (Simpson LVEF, E/e′), making diastolic grading less sensitive, while speckle tracking can unmask subclinical LV/RV dysfunction when image quality permits. The specifics of limitations and pertinent parameters that could be useful in assessing these patients are listed below.

#### 5.2.1. Impact of Lung Hyperinflation and Thoracic Mechanics

Poor echocardiographic windows in patients with COPD, especially the emphysematous phenotype, pose a significant challenge in obtaining clear images and accurate measurements of cardiac dimensions and function, largely due to hyperinflation, and hinder the detection of subtle ventricular dysfunction [55]. The mechanical compression of the heart and altered cardiac geometry due to hyperinflated lungs, particularly during expiration, lead to a smaller cardiac cross-sectional area on dynamic ventilation CT that correlates with airflow limitation [56]. Hyperinflation elevates intrathoracic pressure, imposes a mediastinal constraint, reduces venous return and LV preload, flattens the IV septum and perturbs biventricular filling [57], especially during expiration [19,56]. COPD is associated with smaller chamber sizes and functional alterations; population imaging links emphysema to reduced LV volumes and impaired filling on MRI [19,58].

COPD also alters cardiac morphology and position, producing smaller chamber volumes and clockwise shifts in ECG axes, changes that can mask LV hypertrophy or mislead standard cardiac assessments if pulmonary mechanics are not considered [19,58]. These mechanics help explain echo limitation, namely foreshortening, TRV misalignment, and load-dependent diastolic indices, setting up the need for multi-parametric assessment and, when feasible, advanced imaging [55,56,57,58,59,60]. Left ventricular ejection fraction (LVEF) has limited interpretation when the LV shape is altered and may not be able to reveal/reflect subclinical LV dysfunction.

#### 5.2.2. Challenges in Detecting Subclinical Myocardial Dysfunction

In clinically stable COPD with preserved LVEF/TAPSE (tricuspid annular plane systolic excursion), standard indices can appear “normal” despite impaired mechanics [61]. Ventricular deformation, strain and strain rate cannot be evaluated well with conventional echocardiography. Speckle tracking echocardiography (STE) detects the attenuation of LV-GLS and RV-GLS in COPD—even with only moderate airflow limitations—while LVEF changes are small; meta-analytic data show large effects on GLS and medium-to-large effects on TAPSE, supporting STE for early myocardial involvement [62,63]. 3D echo and CMR also provide more reliable reference quantitation when available [64].

Cardiac MRI (cMRI) could be considered the gold standard imaging modality for the assessment of biventricular function in patients with poor echocardiographic windows, quantifying valvular regurgitation while also offering insight into the pathophysiology owing to its ability to quantify fibrosis, edema and myocardial viability. In cor pulmonale, cMRI can better assess RV dysfunction and fibrosis. Despite this, little work has been done to define a role for the routine use of cMRI in the management of heart failure in the setting of COPD, and there are no published guidelines for its use in this population. Challenges facing the routine use of cMRI in patients with heart failure and COPD include cost, long image acquisition time and the need for respiratory stability and frequent breath holds to guarantee image quality [65,66].

#### 5.2.3. Subclinical Coronary Artery Disease (CAD) in Patients with COPD

Patients with COPD often have a heightened risk for both occult and clinically apparent CAD. Even subclinical CAD in patients with COPD is associated with increased cardiovascular and non-cardiac morbidity, while cardiovascular disease is the leading cause of death in patients with COPD. Patients with COPD frequently require non-contrast-enhanced CT imaging of the chest, and many patients have serial scans, making the CT quantification of coronary calcium a clear choice for screening in this population. The need for functional imaging assessment for myocardial ischemia or assessment for acute CAD (e.g., stress echocardiography, stress myocardial SPECT imaging, dobutamine echocardiography or dobutamine cardiac MRI) should be based on clinical suspicion and driven by current published guidelines. Adenosine and Dipyridamole stress tests are avoided given the risk of bronchospasm [67].

#### 5.2.4. Challenges in Detecting Pulmonary Hypertension in COPD

Complex right ventricular (RV) geometry; the RV’s crescentic, anterior/retrosternal configuration challenges 2D geometric assumptions and can underestimate size/dysfunction and detection of PH. Feasibility is limited: a measurable TR jet is often absent in advanced lung disease, and even when present, echo-derived pulmonary pressures are frequently misclassified in comparison to right heart catheterization (RHC) [55]. Chronic RV pressure overload remodels the ventricle and subvalvular apparatus, displacing the papillary muscles and tethering the leaflets to prevent proper leaflet coaptation. This results in apical tenting and eccentric TR jets that are difficult to align and can either lead to the underestimation of right ventricular systolic pressure (RVSP) or yield no measurable TRV [68]. Commonly used echocardiographic variables and some newer parameters of utility in patients with COPD and co-morbid CHF are listed in Table 2.

Hyperinflation degrades acoustic windows and Doppler alignment, while respiratory pressure swings fragment or shift the TR envelope; eccentric jets and free TR further bias RVSP [69]. In addition, IVC-based right atrial pressure (RAP) assignment is variably accurate in COPD, especially in patients with severe TR, and should not be used in isolation, as RAP error directly propagates into calculated sPAP [69]. The most recent European Society of Cardiology recommendations suggest using TRV > 3.4 m/s to indicate a high probability of having PAH [70]. While in clinical practice, echocardiogram continues to be the main way of screening for PH, it has lower sensitivity; a low RVSP or low-probability study does not reliably exclude PH, so the best practice is to report probability using multiple echo signs and to maintain a relatively low threshold to proceed to RHC for confirmation/phenotyping when clinical suspicion or management stakes are high [69,71].

#### 5.2.5. Echocardiographic Endotypes and Outcomes in COPD with Co-Morbid CHF

The underfilling of the LV in patients with COPD can be attributed to hyperinflation and or reduced pulmonary venous return or the abnormal relaxation of the thickened LV wall from co-morbid HFpEF. Looking at more than 1700 echocardiographic profiles of COPD patients from the COPD and systemic consequences and co-morbidities network (COSYCONET), Abdo et al. identified an echocardiographic endotype of small LV, normal LA size and LV filling pressure (normal E/e′), normal serum troponin and mildly elevated NT-proBNP (compared to HFpEF). When compared to patients with HFpEF and those with normal cardiac function, these patients had higher overall mortality at 4.5 years even after an adjustment for the severity of COPD and exacerbation frequency [72]. The reason for the smaller size of the LV is not well understood, but other reasons like cardiac sarcopenia and reduced LV filling-associated pulmonary HTN may also explain the grim outcomes associated with this endotype.

Another potentially useful approach to the assessment and management of heart failure in patients with COPD is to categorize them based on the presence or absence of significant left or right ventricular dysfunction (Table 3).

#### 5.2.6. Early Detection of AF in Patients with COPD

A lower peak atrial longitudinal strain (PALS) in the four-chamber view can help identify fibrosis, which has been shown to predict the development of AF in patients with COPD, which can then be detected by earlier loop recorder implantation or periodic Holter monitoring [73].

## 6. Unique Issues in Management of Co-Morbid CHF in Patients with COPD

### 6.1. Safety of Beta-Blocker Therapy in COPD with Co-Morbid CHF

It is generally believed that beta-blockers are safe to use in patients with COPD, although RCTs have largely excluded patients with heart failure due to reduced ejection fraction (HFrEF). Recently, a large Swedish registry of patients with COPD and co-morbid HFrEF showed that beta-blocker use was associated with a reduction in a composite outcome of cardiovascular death and hospitalizations for heart failure without compromising COPD exacerbations [74]. Perhaps the main reluctance to use beta-blockers stems from the presence of associated asthma, especially the late-onset type, in elderly patients [46]. Fixed obstruction on spirometry with incomplete or no bronchodilator response can point more toward COPD, and in such situations, withholding beta-blockers in patients with co-morbid CHF has been shown to be harmful [75].

Earlier studies showed that beta-blockers can reduce mortality and exacerbations related to COPD [76], but this was not confirmed in an RCT [77]. Selective beta-1 blockers should be used to treat approved cardiovascular indications in patients with COPD but not for the purpose of preventing COPD exacerbations [78,79]. However, in patients with HF, the beta-1’s cardio-selectivity inversely decreases as the therapeutic dose is maximized [80]. Although carvedilol is among the most preferred beta-blockers used in patients with CHF (especially non-ischemic HFrEF), its beta-1 and -2 blocking effects have led it to be left out of most clinical trials. Limited data affirm its safe use in patients with COPD with co-morbid CHF [81,82].

#### Concomitant Use of Beta-Blockers with Bronchodilators

Recent trials have confirmed the safety of long-acting muscarinic antagonists (LAMAs) and long-acting beta agonists (LABAs) as the mainstay of treatment in COPD patients with high cardiovascular risk [83,84,85,86]. Of note, the mortality reduction in both the ETHOS and IMPACT trials has been attributed to decreased cardiovascular deaths [87,88], and in ETHOS, it was noted to be more pronounced in patients with a higher baseline eosinophil count > 200 microL. In fact, a post hoc analysis showed a lower incidence of MACE when ICS was included in the therapy (1.4% vs. 2.1%) [89]. A single-center RCT showed that albuterol improved pulmonary vasodilation and cardiopulmonary reserve in patients with HFpEF during exercise without increasing pulmonary venous pressures, although no hard clinical outcomes were reported [90]. More studies are needed to test the potential benefits of protection from cardiovascular events, the mechanisms involved and all-cause mortality with combination bronchodilators and the relation to the reduction in the rate of exacerbations. The most recent GOLD report recommends continuing beta-blockers in patients with CVD, while a patient is on chronic LAMA/LABA therapy.

### 6.2. The Use of Non-Invasive Ventilation (NIV) in the Management of COPD with Co-Morbid CHF

In COPD, NIV provides support for ventilation and mitigates air trapping, hyperinflation and auto-PEEP from dynamic airway collapse. In acute heart failure, NIV helps prevent alveolar collapse from pulmonary edema via PEEP and improves gas exchange while also improving cardiac output (CO) by decreasing both LV preload and after load [91]. These physiological effects translate into significant benefits at the bedside. For instance, there is substantial and strong evidence of improved hospital mortality and intubation rates in acute hypercapnic exacerbations of COPD treated with bilevel PAP [92] and in acute cardiogenic edema treated with either continuous or bilevel PAP [93]. There is also strong recent evidence that the home use of high-pressure support with a backup rate improves mortality and quality of life in those with chronic hypercapnic COPD [94].

Whether the use of home NIV with high-pressure support can be safely extended to patients with chronic hypercapnic COPD with co-morbid heart failure is not entirely clear. The presence of cardiac disease was an exclusion in some of the trials of NIV in COPD [94], and the potential adverse cardiac effects were not assessed in a major RCT which used high-pressure NIV therapy in COPD [95]. High-intensity pressures were reported to reduce CO by 15% when compared to low-intensity pressure in a short-term daytime trial [96]. In a more representative, longer-term, randomized crossover study of the cardiac effects of high- vs. low-intensity NIV in chronic hypercapnic COPD, high-intensity ventilation generally did not have adverse effects on cardiac output, except potentially in patients with co-morbid heart failure for whom the authors recommended regular cardiac monitoring [97]. Our approach would be to consider high-intensity NIV in COPD and co-morbid heart failure but with regular cardiac monitoring. One potential caveat is the use of NIV in those with hypotension and right-sided heart failure, who may be particularly susceptible to the adverse effects of NIV [91].

### 6.3. Future Directions

The role, if any, that NT-proBNP will have in screening for CHF in patients with COPD needs to be better defined. It has been shown that NT-proBNP may not be elevated in the small LV phenotype. This topic becomes even harder to study when conventional echocardiography has inherent limitations in the early detection of LV underfilling in patients with COPD and may itself be late in confirming the diagnosis of CHF. The phenotyping and imaging-based endotyping of patients at risk for CHF related to COPD can help with earlier identification, strategies for screening and the better management of patients with co-morbid COPD and CHF. A better understanding of the pathogenesis of the small LV phenotype and its association with severely decreased lung function and mortality is needed. Recent studies with triple therapy compared to LAMA/LABA have shown improved overall mortality benefits, but the evidence for cardiovascular event reduction is very limited. Given the high risk and burden for cardiovascular events, more studies using inhaled interventional therapies are required that can lead to a reduction in cardiovascular event risk.

## Figures and Tables

**Table 1:** Outcomes in COPD with co-morbid CHF.

	Study Author (Year, Type)	Inclusion Criteria (*n*)	Outcomes	Major Limitations
1.	Dai et al. [10] - 2020 - Retrospective - Single-center	1st adult ICU admission - Without COPD or CHF, *N* = 20,507 - With COPD only, *N* = 1575 - With CHF only, *N* = 6190 - With COPD and CHF, *N* = 1317	With COPD and CHF versus without COPD or CHF - 28-day mortality: [1.55 (1.33–1.80); *p* < 0.001] - 90-day mortality: [1.64 (1.46–1.85); *p* < 0.001]	- Retrospective. - ICD-9 codes used for identifying COPD and HF (no strict definitions of COPD and HF). - Single-center data, limited generalizability.
2.	Gulea et al. [9] - 2021 - Retrospective - OptumLabs Data Warehouse	COPD with - HFpEF, *N* = 3843 - HFmEF, *N* = 562 - HFrEF, *N* = 1014	HFmEF vs. HFpEF//HFrEF vs. HFpEF - 1-year all-cause hospitalization: [1.01 (0.88–1.16); *p* = 0.888]//[1.07 (0.96–1.20); *p* = 0.224] - HF-specific hospitalization: [1.03 (0.81–1.32); *p* = 0.799]//[1.54 (1.29–1.84); *p* < 0.001] - Acute exacerbation of COPD: [0.82 (0.69–0.97); *p* = 0.024]//[0.75 (0.65–0.97); *p* < 0.001] - Mortality (27 months after HF diagnosis): [0.99 (0.84–1.16); *p* = 0.867]//[1.16 (1.03–1.32); *p* = 0.019]	- Retrospective. - Study unable to distinguish improved EF due to lack of data. - Undocumented spirometry; diagnosis of COPD was based on validated ICD codes and COPD-related medications. - Overdiagnosis of COPD because of underlying factors, but undiagnosed HF cannot be ruled out. - Unable to capture prescriptions not submitted to insurance, leading to a potential underestimation of prescription rates. - Analyses not adjusted for the duration or severity of COPD. - Mortality not accounted for as a competing risk for hospitalization beyond 1 year. - Potential for residual confounding.
3.	Ehteshami-Afshar et al. [11] - 2021 - Randomized clinical trial	HF patients with NYHA II-IV, LVEF ≤ 40% (amended to ≤35% during study) and BNP ≥ 150 or NT-proBNP ≥ 600 pg/mL - With COPD, *N* = 1080 - Without COPD, *N* = 7319	HFrEF with COPD vs. without COPD - Composite of cardiovascular death of first HF hospitalization: [1.18 (1.05–1.34); *p* = 0.007] - HF hospitalization: [1.32 (1.13–1.54); *p* < 0.001] - Cardiovascular hospitalization: [1.45 (1.29–1.64); *p* < 0.001] - All-cause hospitalization: [1.40 (1.32–1.49); *p* < 0.001] - All-cause death: [1.14 (0.99–1.31); *p* = 0.080] - Mean change in KCCQ clinical symptom score: minus 6.26 vs. minus 3.43 (*p* < 0.001)	- Investigator-derived diagnosis of COPD. - No requirement for investigator documentation for previous smoking history. - Lacking data on spirometry and possible misdiagnosis. - Recruitment bias leading to exclusion of some individuals with severe pulmonary disease. - No adjustment for multiplicity.
4.	Mooney et al. [12] - 2021 - Randomized clinical trial	Chronic symptomatic HFpEF (LVEF ≥ 45%), NYHA II-IV, with elevated NT-proBNP - With COPD, *N* = 670 - Without COPD, *N* = 4121	HFpEF with COPD vs. without COPD - Composite of total HF hospitalizations and cardiovascular deaths: [1.51 (1.25–1.83); *p* < 0.001] - Total HF hospitalizations: [1.54 (1.24–1.90); *p* < 0.001] - Cardiovascular death: [1.42 (1.10–1.82); *p* = 0.006] - Non-cardiovascular death: [1.67 (1.23 (2.27); *p* < 0.001] - All-cause death: [1.52 (1.25–1.84); *p* < 0.001] - Mean change in KCCQ clinical symptom score: minus 5.49 vs. minus 1.52 (*p* < 0.001)	- Investigator-derived diagnosis of COPD. - No prespecified diagnosis criteria in the protocol. - Since spirometry was not a requirement, likely underdiagnosis of COPD is possible. - Exclusion of patients with severe pulmonary disease.
5.	Xu et al. [13] 2025 Meta-analysis	COPD with HFpEF *N* = 11 studies; 18,602 participants	HFpEF with COPD vs. without COPD - Adverse outcomes: [1.84 (1.35–2.51); *p* < 0.001] - Mortality: [1.62 (1.34–1.95); *p* < 0.001] - Hospitalization-associated outcomes: [1.66 (1.47–1.87); *p* < 0.001]	- COPD defined by clinical records, which may be based on medication history, previous physician’s diagnosis or spirometry. - Two studies identified as moderate-quality by NOS quality assessment system. - Language bias: only studies reported in English were included. - Limited generalizability since the research population did not have a good coverage of different ethnic groups. - Only two to three data points were included in each subgroup analyses, which is relatively insufficient.
6.	Sandoval-Luna et al. [14] - 2024 - Prospective cohort	Adults with clinically diagnosed HF - With COPD, *N* = 441 - Without COPD, *N* = 2073	HF with COPD vs. without COPD: - All-cause mortality at ≥6-month follow-up: [1.47 (1.02–2.11); *p* < 0.001]	- Study did not include data on COPD severity or treatment. - Several confounders such as body mass index or other relevant diagnoses (e.g., asthma). - Study did not include data on echocardiographic variables from all patients, which could be a confounding factor.
7.	Hesse et al. [15] - 2022 - EHR data from 2 hospitals in the UK	Patients admitted with an exacerbation of COPD - With known HF, *N* = 94 - New diagnosis of HF, *N* = 38 - No HF, *N* = 344	Survival analysis of COPD-HF patients - COPD with vs. without HF, death at 1l.7 months: 58.3% with HF and 31.4% without HF [2.03 (1.46–2.82); *p* < 0.001] - More deaths at 7.9 months in patients with known HF than those with newly diagnosed HF (61.7% vs. 50.0%; *p* = 0.079)	- Only patients with confirmed COPD and echocardiography-diagnosed HF were included, likely leading to an underdiagnosis of HF.
8.	Becher et al. [16] - 2025 - Retrospective - Swedish HF registry	Clinician-judged HF - With COPD, *N* = 12,355 - Without COPD, *N* = 85,549	Prevalence, COPD in overall HF cohort, 13%, Prevalence, COPD in HFrEF, HFmrEF, HFpEF = 11%, 12%, 16% respectively HF with COPD vs. without COPD 3. Composite of cardiovascular death and first HF hospitalization: [1.15 (1.11–1.18); *p* < 0.001] 4. Cardiovascular deaths: [1.11 (1.06–1.15); *p* < 0.001] 5. First HF hospitalization: [1.15 (1.12–1.19); *p* < 0.001] 6. All-cause death: [1.33 (1.30–1.37); *p* < 0.001] 7. Non-cardiovascular deaths: [1.71 (1.64–1.78); *p* < 0.001]	- Observational study design. - Unmeasured and unknown confounders, preventing this study from assessing a causal relationship between COPD and outcomes. - Limited coverage of SwedeHF and the national setting, limiting the generalizability of this study. - COPD diagnosis based on ICD-10 codes, likely capturing a clinician-judged diagnosis (spirometry data not captured). - Study prone to over- and under-coding since it was based on ICD-10 codes.
9.	Crisafulli et al. [17] - 2023 - Retrospective, cross-sectional study - REPOSI registry, Italy	≥65 years age - With COPD, *N* = 1154 - With HF, *N* = 813 - With COPD+HF, *N* = 376	COPD+HF vs. COPD alone was an independent predictor for increased risk of death at 1-year: [1.74 (1.16–2.61); *p* < 0.001] - Significantly more deaths in COPD+HF cohort (Χ2 = 7.74 and 9.69 at 6 months and 1 year of follow-up respectively) - COPD+HF had higher all-cause mortality (*p* = 0.010), mortality for respiratory causes (*p* = 0.006), mortality for cardiovascular disease causes (*p* = 0.046), and mortality for respiratory and cardiovascular causes (*p* = 0.009)	- Retrospective study derived from a dataset registry. - Relatively short follow-up duration (one year). - Lack of precise spirometric data. - Likely overdiagnosis of COPD based on clinical findings and historical information.
10.	Dewan et al. [18] - 2021 - Randomized clinical trial	Adults with LVEF ≤ 40%, NYHA II-IV, and elevated NT-proBNP - With COPD, *N* = 585 - Without COPD, *N* = 4159	HFrEF with COPD vs. without COPD - Composite of worsening HF or cardiovascular death: [1.44 (1.21–1.72); *p* < 0.001] - Worsening HF event: [1.74 (1.41–2.15); *p* < 0.001] - First HF hospitalization: [1.78 (1.44–2.20); *p* < 0.001] - Cardiovascular death: [1.28 (1.00–1.63); *p* = 0.049]	- Small proportion of COPD patients compared to those without. - Investigator-reported diagnosis of COPD. - Likely under-representation of COPD since spirometry was not performed on all patients. - Study participants were selected for a randomized controlled trial and were probably “healthier” than real-world patients. - Likely exclusion of severe COPD patients, since investigators were asked to not include patients with a life expectancy of <2 years.

**Table 2:** Echocardiographic variables of significance in patients with COPD and co-morbid CHF.

Parameter	Cut-Off Value	Notes
Tricuspid regurgitation velocity (TRV)	>2.8–3.4 m/s	Screening cue for elevated sPAP. Sensitivity—43–67%; specificity—75–83%. Can be underestimated in eccentric jets.
Systolic pulmonary artery pressure (sPAP)	≥35–40 mm Hg	Positive screen criteria for PH in stable COPD but often underestimated by >10 mmHg.
Tricuspid annular plane systolic excursion (TAPSE) (RV longitudinal systolic function)	<16 mm (or 1.6 cm)	TAPSE < 16 mm indicates RV systolic dysfunction and is an unfavorable prognostic threshold associated with mortality. A value of <1.8 cm was used as a criterion for a positive screen for PH in stable COPD outpatients.
RV dilation (RV basal diameter)	>42 mm	RV dilation is a criterion for a positive screen for PH in stable COPD outpatients. RV dilatation is associated with all-cause mortality. An RV basal end-diastolic diameter > 42 mm suggests pressure/volume overload.
Tricuspid regurgitation pressure gradient (TRPG)	>30–40 mmHg	Sensitivity—~55%. Patients with >40 mmghg were found to have an increased risk of mortality and decreased exercise tolerance.
RV S′ (systolic velocity of the lateral tricuspid valve annulus)	<9.5 cm/s	Decreased tricuspid annular systolic velocity (S′) is associated with increased all-cause mortality in COPD patients. Sensitivity/specificity for RV dysfunction ~90%/85%.
RV myocardial performance index or Tei index	>0.55	Indicates global RV systolic and diastolic dysfunction, inaccurate during Afib.
TAPSE/PASP (RV–PA coupling)	≤0.36 mm/mmHg	Lower values indicate RV-PA uncoupling and worse prognosis and predicts mortality.
Right ventricular free wall longitudinal strain (RVFWLS)	<−20%	Has 95.8% sensitivity and 88% specificity for detecting early right ventricular dysfunction. Measured by speckle tracking.
Septal flattening/D-shaped LV	Presence/increased eccentricity index	Indirect indicators of RV pressure overload and PH.
Mitral E/e′ ratio	>8 (rest), >15 (stress)	Index of mean LV filling pressure. Associated with increased mortality. Load-dependent.

**Table 3:** Management aspects based on ventricular function in COPD patients with co-morbid CHF.

Normal LV and RV function Best prognosis Safer use of nitrates and central calcium channel blockers when needed Beta-blocker indication weakest here Little fear related to steroid and diuretic use	Reduced LV and normal RV function Heart failure probably the bigger issue here, so heavily emphasize diuresis and guideline-directed medical therapy, which is likely to improve COPD by relieving pulmonary congestion Beta-blockers more important here, central calcium channel blockers contraindicated
Normal LV and reduced RV function COPD likely to be the bigger issue here, so emphasize aggressive COPD management, which is likely to improve cardiac function through more optimal ventricular filling Need to be cautious about the use of venodilators like nitrates and need to avoid aggressive diuresis due to preload-dependent RV Lowest threshold for right heart catheterization here	Reduced LV and reduced RV function Poorest prognosis, likely to have advanced heart and lung disease Consideration for advanced heart failure consultation here with thoughts of either palliation or assessment for double-organ transplant candidacy

## Data Availability

No new data were created or analyzed in this study.
